# Epileptiform EEG discharges and sevoflurane in children: Protocol of a systematic review and meta-analysis

**DOI:** 10.1097/MD.0000000000017401

**Published:** 2019-10-04

**Authors:** Mengrong Miao, Yuehua Xu, Xuhui Cong, Liyuan Zhang, Jiaqiang Zhang

**Affiliations:** a Department of Anesthesia and perioperative medicine, Henan University People's Hospital, Henan Provincial People's Hospital of Henan University; b Department of Anesthesia and perioperative medicine, Henan Provincial People's Hospital; c Department of Surgery, First Hospital of Henan Provincial People's Hospital, Zhengzhou, Henan, China.

**Keywords:** Children, EEG, meta-analysis, seizures, sevoflurane, spike

## Abstract

**Background:**

Epileptiform discharges in electroencephalogram (EEG) have been frequently reported in children undergoing sevoflurane mask induction. However, the incidence, characteristics and risk factors of these epileptiform patterns during sevoflurane anesthesia in children are poorly understood. The aim of this study is to systematically review the epileptic potential of sevoflurane in children with the EEG monitoring.

**Methods:**

PubMed, EMBASE, Cochrane library (Central) will be systematically searched from inception to December 2018. The effect of sevoflurane on epileptic EEG patters in children will be studied. The primary outcome will be the incidence of epileptic discharges, the characteristics and risk factors of these epileptic discharges. Meta-analysis will be calculated using R software 3.5.1.

**Results:**

This study will offer new evidence of the incidence, characteristics and risk factors of EEG epileptic discharges during sevoflurane anesthesia.

**Conclusion:**

The conclusion drawn from this systematic review will benefit the children with or without epilepsy undergoing sevoflurane anesthesia.

**Ethics and dissemination:**

Ethics approval is unnecessary because data of individual patients will not be included and no privacy will be involved. The results of this review will be published in a peer-reviewed journal or a conference report. Amendments of the basic protocol will be documented in the comprehensive review.

**PROSPERO registration number:**

PROSPERO CRD 42019122008.

## 1 Introduction

Sevoflurane is a widely used inhalation anesthetics in children for its hemodynamic stability and lack of respiratory irritation. But the epileptic potential of sevoflurane was frequently confirmed in children during anesthesia induction.^[1–5]^ In a trial conducted by Tanaka et al, age-adjusted 1.5 minimum alveolar concentration (MAC) sevoflurane was associated with increased spikes per lead and increased number of leads with spikes in electrocorticogram in children with epilepsy compared with sevoflurane at lower concentration (2.5%).^[6]^ The epileptic potential of sevoflurane can also be observed even in the pediatric patients without epilepsy. Two children showed generalized convulsive movements and board-like rigidity during induction of 7% to 8% sevoflurane were early reported by Zacharias in 1997.^[7]^ Vakkuri et al found epileptic EEG abnormalities in children without epilepsy during mask induction of 8% sevoflurane.^[1]^

The epileptic potential of sevoflurane was usually characterized by epileptiform discharges in Electroencephalogram (EEG). According to the description of Vakkuri et al,^[1]^ the epileptiform discharges can be divided into several types: delta with spikes (DSP), rhythmic polyspikes (PSR), periodic epileptiform discharges (PED), and suppression with spikes (SSP). Many studies have shown that these paradoxical discharges found in children with or without epilepsy are related to poorer cognitive performance^[8]^ and emergency delirium.^[9]^ However, the incidence, prevention and risk factors of these epileptiform patterns during sevoflurane anesthesia in children are still under debate. Based on the above facts and the existing studies, it is urgent to conduct this systematic review and meta-analysis to evaluate the epileptic potential of sevoflurane in EEG during general anesthesia in children.

## 2 Methods

### 2.1 Design and registration of the review

This protocol was registered in the international prospective register of systematic reviews (PROSPERO, CRD42019122008) and will be conducted according to the Preferred Reporting Items for Systematic Reviews and Meta-Analyses Protocols (PRISMA-P) guidelines.^[10]^

### 2.2 Criteria for including studies in this review

#### 2.2.1 Types of studies

The randomized control trials (RCTs) will be included into this systematic review. The controlled clinical trials (CCTs), observational studies such as case-control, cross-section, and cohort studies, retrospective analysis will also be included into this study if there is no RCT.

#### 2.2.2 Types of participants

Pediatric patients (<18 years) undergoing sevoflurane induction with EEG monitoring will be included into this study.

#### 2.2.3 Types of interventions

Other drug or none control group will be compared with sevoflurane.

#### 2.2.4 Types of outcome measures

The primary outcome will be the incidence of epileptic discharges during sevoflurane induction.

The secondary outcomes will include the incidence of different types of epileptiform discharges, the characteristics, risk factors and the prevention of these epileptic discharges, and other adverse events such as convulsive movements.

### 2.3 Search methods for identification of studies

PubMed, EMBASE, Cochrane library (central) will be systematically searched by 2 authors from inception to December 2018. The references of included studies, relevant systematic reviews will also be screened for additional eligible studies. We will contact the original authors to obtain unpublished data of the included studies. No restrictions on the language of the included literatures will be applied. A complete search strategy of PubMed is available in Table 1.

**Table 1:**
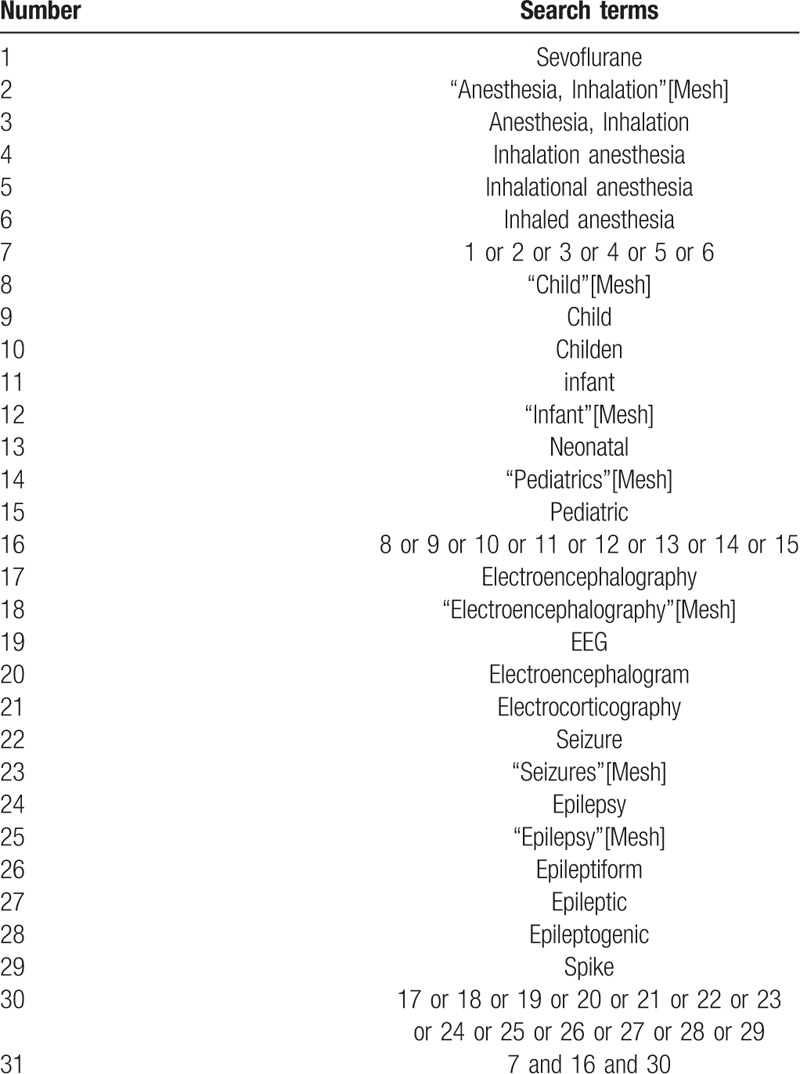
Search strategy applied in PubMed.

### 2.4 Data collection and analysis

#### 2.4.1 Selection of studies

Two reviewers will independently screen the titles and abstracts of the searched literatures for identifying potential studies. Full-texts of relevant studies will be obtained to check, if it is unclear for inclusion from title and abstract. All of the included literatures will be confirmed by full-text review. Discrepancies will be resolved by discussion between authors.

#### 2.4.2 Data extraction and management

Researchers will use a standard data collection form to extract study characteristics and outcome data of the included literatures. Two authors will extract the data independently and check each other for accuracy. The following data of the selected studies will be extracted:

Participants characteristics: mean age, sex, ASA class; Trial characteristics: author name, year of publication, study design; Intervention characteristics: sevoflurane concentration, intervention, premedication, adverse events, the type of EEG monitoring, primary outcome and secondary outcome. Any discrepancies will be resolved by discussion between authors. We will contact the original authors for more essential information.

#### 2.4.3 Assessment of risk of bias

Different Methodological quality assessment tools will be used according to the types of the included studies by 2 authors. The revised Jadad scale^[11]^ will be used to assess the risk of bias for randomized clinical trials. The Newcastle–Ottawa scale^[12]^ will be used to assess the risk of bias of controlled clinical trials (CCTs), case-control studies, cohort studies, and retrospective analysis. And the Agency for Healthcare Research and Quality (AHRQ) tool^[13]^ will be used to assess the risk of bias for cross-sectional study. We will settle all disagreements by negotiation.

#### 2.4.4 Data synthesis and statistical analysis

Calculation of pooled prevalence will be performed using R software 3.5.1. Data that cannot be synthesized will be described qualitatively. Meta-analysis will be performed using a random-effects model accounting for clinical heterogeneity. I^2^ test will be carried out to measure the heterogeneity across studies. A study will be considered significant heterogeneity if I^2^ > 50%. A *P* value of less than .05 will be considered statistically significant. We will draw a funnel plots to detect the potential reporting biases if at least 10 studies are included. The Egger test will be applicated to determine the symmetry of the funnel plot.

#### 2.4.5 Sensitivity analysis and subgroup analysis

We will perform a sensitivity analysis by excluding trials with high risk of bias. Furthermore, we will also conduct a sensitivity analysis by excluding each included trial to investigate the robustness of the findings. In addition, subgroup analyses will be performed to explore the heterogeneity of meta-analyses if the data is available.

## 3 Discussion

Sevoflurane is often associated with epileptic EEG activity during induction in children.^[1]^ Some studies had shown that lower sevoflurane concentration during induction and shorter induction time can reduce the occurrence of epileptic discharge.^[4,14]^ Iijima et al reported that nitrous oxide may diminish epileptogenic effects of sevoflurane in patients with epilepsy.^[5]^ And the addition of alfentanil in the induction process of sevoflurane may have a protective effect on sevoflurane induced epileptic discharges.^[2]^ As we know, the current study will be the first meta-analysis for the pooled prevalence and the prevention of epileptiform discharges during sevoflurane mask induction in children, and will firstly offer the systematic evidence for the effect of sevoflurane on epileptic EEG in children.

## Author contributions

**Conceptualization**: Mengrong Miao, Xuhui Cong, Liyuan Zhang, Jiaqiang Zhang

**Data curation**: Mengrong Miao, Yuehua Xu, Jiaqiang Zhang

**Formal analysis**: Mengrong Miao, Yuehua Xu, Jiaqiang Zhang

**Methodology**: Yuehua Xu, Liyuan Zhang, Liyuan Zhang,

**Project administration**: Mengrong Miao, Yuehua Xu

**Software**: Yuehua Xu, Liyuan Zhang, Liyuan Zhang,

**Supervision**: Xuhui Cong, Liyuan Zhang, Jiaqiang Zhang

**Writing – original draft**: Mengrong Miao

**Writing – review & editing**: Mengrong Miao, Liyuan Zhang, Jiaqiang Zhang
